# Multi-omics profiling of TGF-β isoforms and regulatory miRNAs in astrocytic tumors reveals TGF-β-3 as a prognostic biomarker

**DOI:** 10.3389/fonc.2025.1592685

**Published:** 2025-06-20

**Authors:** Klaudia Skóra, Damian Strojny, Dawid Sobański, Rafał Staszkiewicz, Kamil Bryś, Paweł Gogol, Krzysztof Bereza, Michalina Masternak, Beniamin Oskar Grabarek

**Affiliations:** ^1^ Department of Neurological Rehabilitation, District Hospital of St. Padre Pio in Sędziszów Małopolski, Sędziszów Małopolski, Poland; ^2^ Collegium Medicum, WSB University, Dąbrowa Górnicza, Poland; ^3^ Department of Neurology, New Medical Techniques Specialist Hospital of St. Family in Rudna Mała, Rzeszow, Poland; ^4^ Institute of Health Care, National Academy of Applied Sciences in Przemyśl, Przemyśl, Poland; ^5^ Department of Neurosurgery, St. Raphael Hospital, Krakow, Poland; ^6^ Department of Neurosurgery, 5th Military Clinical Hospital with the SP ZOZ Polyclinic in Krakow, Krakow, Poland; ^7^ Department of Neurosurgery, Faculty of Medicine in Zabrze, Academy of Silesia in Katowice, Katowice, Poland; ^8^ Department of Anesthesiology and Intensive Care, Our Lady of Perpetual Help Hospital in Wołomin, Wołomin, Poland; ^9^ Department of Trauma and Orthopedic Surgery, Our Lady of Perpetual Help Hospital in Wołomin, Wołomin, Poland; ^10^ Pain Treatment Clinic, Our Lady of Perpetual Help Hospital in Wołomin, Wołomin, Poland; ^11^ Department of Mother and Child Health, Faculty of Health Sciences, Institute of Nursing and Midwifery, Jagiellonian University Medical College, Krakow, Poland; ^12^ Department of Brachytherapy, Jagiellonian University Medical College, Krakow, Poland; ^13^ Silesian Center for Rehabilitation and Manual Therapy Revita in Mysłowice, Mysłowice, Poland; ^14^ Faculty of Medicine and Health Sciences, Andrzej Frycz Modrzewski University, Cracow, Poland

**Keywords:** transforming growth factor beta 1-3, astrocytic tumors, survival analysis, micro RNA, molecular marker

## Abstract

**Background:**

Astrocytic tumors, particularly glioblastomas, are aggressive brain malignancies with poor prognosis. Transforming growth factor-beta (TGF-β) isoforms—TGF-β-1, TGF-β-2, and TGF-β-3—play critical roles in glioma progression, yet their isoform-specific expression patterns and regulatory mechanisms remain incompletely defined. This study aimed to evaluate the differential expression of TGF-β isoforms and their regulation by epigenetic mechanisms and microRNAs (miRNAs) across astrocytic tumor grades.

**Methods:**

Sixty-five astrocytic tumor samples (WHO grades 2-4) were analyzed. Gene and protein expression of TGF-β-1, -2, and -3 were assessed using reverse transcription quantitative polymerase chain reaction (RT-qPCR), enzyme-linked immunosorbent assay (ELISA), and immunohistochemistry (IHC). Promoter methylation was analyzed using methylation-specific PCR (MSPCR). Differentially expressed regulatory miRNAs were identified by microarray and in silico target prediction. Survival associations were evaluated by Kaplan–Meier and Cox regression analyses.

**Results:**

TGF-β-1 and TGF-β-3 were significantly upregulated in high-grade astrocytomas (p < 0.05), whereas TGF-β-2 showed no consistent changes. TGF-β-3 expression strongly correlated with poor survival (Exp(B) = 1.02644, p < 0.0001), while TGF-β-1 showed a weaker, non-significant association. Among regulatory miRNAs, hsa-miR-2278 (targeting TGF-β-3) was upregulated and significantly associated with worse survival (Exp(B) = 1.437, p = 0.008), while hsa-miR-3196 (targeting TGF-β-1) was downregulated and trended toward better prognosis (Exp(B) = 0.8897, p = 0.076).

**Conclusion:**

TGF-β-3 is a potent prognostic biomarker in astrocytic tumors and a promising candidate for targeted therapeutic intervention. Regulatory miRNAs such as hsa-miR-2278 and hsa-miR-3196 may serve as molecular modulators of TGF-β signaling and potential adjuncts in personalized glioma therapy. These findings warrant further investigation into miRNA-based therapeutics targeting the TGF-β axis in high-grade gliomas.

## 1 Introduction

Astrocytic tumors, originating from astrocytes, are among the most prevalent brain tumors and are classified into four grades based on histological characteristics and severity (1–3). Despite advances in clinical research, the prognosis for these tumors remains poor. Patients with low-grade gliomas (LGGs) (grades II and III) have a median survival of 5–10 years, whereas those with high-grade gliomas (grade IV) typically survive only 1–2 years (4). Glioblastoma multiforme (GBM), the most common and aggressive grade IV astrocytic tumor, is associated with a particularly poor prognosis (5, 6).

The integration of molecular biology into neuro-oncology has significantly refined the classification of brain tumors. The World Health Organization (WHO) revised its classification system in 2016, incorporating genomic profiling and epigenetic changes (7). The latest 2021 WHO classification further underscores the increasing importance of molecular diagnostics, introducing a distinction between grade IV astrocytoma and glioblastoma, both of which were previously grouped under the same category (8–10). As part of this refinement, some tumors previously classified as grade 3 astrocytomas have been redefined as glioblastoma grade 4 based on their molecular features. These advancements highlight the critical role of molecular profiling in diagnosing and classifying astrocytic tumors (8–10).

Molecular biomarkers play a vital role in determining prognosis and guiding treatment strategies (11). Key markers include isocitrate dehydrogenase (IDH1/2) mutations, MGMT promoter methylation, 1p/19q co-deletion, and epidermal growth factor receptor (EGFR) amplification. Among these, IDH mutations are particularly significant, as they are associated with improved survival across all glioma grades. For example, patients with IDH-mutated grade IV astrocytomas have a median survival of approximately 31 months, whereas those with IDH wild-type tumors have a shorter median survival of 15 months (12–14).

Transforming growth factor-beta (TGFβ) is a multifunctional cytokine family that includes six isoforms, three of which—TGFβ1, TGFβ2, and TGFβ3—are present in humans. These isoforms share considerable sequence similarity (71–79%) but are encoded by distinct genes. Their biological activity is highly dependent on the relative expression of each isoform, influencing various cellular processes (15). TGFβ plays a crucial role in both physiological and pathological conditions by regulating key cellular functions such as growth, differentiation, inflammation, and tissue repair (16). As an anti-inflammatory cytokine, TGFβ is secreted by immune cells following injury, modulating immune responses and promoting healing (17). Studies by Cekanaviciute et al. have shown that in response to *Toxoplasma gondii* infection, TGFβ signaling is activated in astrocytes, which helps control neuroinflammation. Conversely, inhibition of TGFβ signaling leads to excessive immune infiltration, increased secretion of pro-inflammatory cytokines and chemokines, and neuronal damage (18). Beyond its immunoregulatory role, TGFβ is integral to nervous system function. Among its isoforms, TGFβ1 is the most abundant and is particularly involved in astrocyte-mediated scar formation following brain injury (19). Experimental studies have demonstrated that TGFβ upregulates neurocan, a chondroitin sulfate proteoglycan that contributes to glial scar development (20). TGFβ signaling is implicated in multiple cancers, including lung, breast, pancreatic, colorectal, and melanoma (21). It has also been extensively studied in gliomas, yet the specific interactions between TGFβ1, TGFβ2, and TGFβ3 isoforms in astrocytic tumors remain poorly understood (22, 23).

TGFβ is also a key player in tumorigenesis, particularly in gliomas. Its role in cancer is complex and often described as the “TGFβ paradox.” (22, 24). In early tumor development, TGFβ functions as a tumor suppressor by inhibiting cell proliferation, inducing differentiation, promoting apoptosis and autophagy, and limiting angiogenesis and inflammation (25). However, in advanced cancer stages, TGFβ facilitates tumor progression by promoting extracellular matrix remodeling, enhancing angiogenesis, and creating an immunosuppressive tumor microenvironment that enables metastasis (24, 26, 27). Clinically, this dual role is significant—elevated TGFβ levels correlate with better prognosis in early-stage tumors but are linked to increased aggressiveness, invasiveness, and poorer outcomes in advanced malignancies. This underscores the potential of TGFβ as both a biomarker and a therapeutic target (28–30).

A study by Naik et al. explored the relationship between epigenetic factors and TGF-β signaling pathways (31). The authors concluded that dynamic epigenetic modifications are essential for determining cancer cell behavior, influencing tumor microenvironment interactions, and affecting the overall carcinogenesis process. Their analysis revealed complex regulatory networks in tumors, involving long non-coding RNAs (lncRNAs), microRNAs (miRNAs), and post-translational histone modifications, all of which are closely linked to TGF-β signaling (31). Similarly, Ding et al. demonstrated that epigenetic regulation of TGF-β, including chromatin remodeling, non-coding RNA regulation, DNA methylation, and histone modifications, not only contributes to tumor cell formation and growth but also affects the response to radiotherapy (32). This study highlights that understanding the impact of epigenetics is a key aspect in both the diagnosis and treatment of cancer patients.

Methylation profiling has emerged as an essential tool in brain tumor classification, complementing histological evaluation for more accurate tumor identification. The integration of histopathology and molecular techniques is expected to enhance prognostic accuracy and improve patient management. In some cases, molecular alterations can justify classifying a tumor as malignant, even if histological features suggest a lower grade. This shift reflects the growing reliance on molecular markers in neuro-oncology and the potential for personalized therapeutic strategies (33).

MicroRNAs (miRNAs) are short, non-coding RNA molecules that play a crucial role in gene expression regulation by blocking or degrading mRNA (34). MiRNAs regulate gene expression by targeting mRNA and binding directly to complementary sites in the 3′ untranslated region (3′ UTR) of the target mRNA (35). As a result, miRNAs can regulate multiple mRNA targets involved in various biological processes, including DNA damage repair, apoptosis, proliferation, cell cycle regulation, senescence, invasiveness, and angiogenesis (36). Experimental studies confirm that several miRNAs act as modulators of TGF-β signaling at multiple levels by targeting ligands, receptors, R-Smad, co-Smad, I-Smad, and non-Smad pathway components, as well as downstream targets of TGF-β signaling (37).

Therefore, the study aimed was to evaluate variances in the expression patterns of TGFβ1–3 in astrocytic tumors with respect to the degree of malignancy.

## 2 Materials and methods

### 2.1 Patient selection and clinical sampling

This study involved the collection of astrocytic brain tumor specimens from 65 patients undergoing surgical resection. The procedures were conducted at two neurosurgical centers in Krakow, Poland: the Department of Neurosurgery at the 5th Military Clinical Hospital with the SP ZOZ Polyclinic and the Department of Neurosurgery at Szpital św. Rafała. Of the 65 patients included in the study, 35 were female and 30 were male, with an average age of approximately 56 to 59 years across different malignancy grades. Among female patients, 10 had G2 tumors, 7 had G3 tumors, and 18 had G4 tumors. In the male group, 7 had G2 tumors, 5 had G3 tumors, and 18 had G4 tumors. To ensure standardized preoperative conditions, all participants followed a fasting regimen, consuming their final meal at 6 PM the evening before surgery. This was in accordance with the hospitals’ nutritional policy, which schedules the last meal at this time to prepare patients for surgery the following morning. All procedures were performed between 8 AM and 11 AM, and only elective surgeries were included in the study.

Eligibility for participation was determined based on predefined criteria. Patients qualified for the study if they were scheduled for astrocytic tumor resection at one of the designated hospitals and provided informed consent. Individuals with additional neoplastic conditions were excluded.

The inclusion criteria were (1): histopathologically confirmed astrocytic brain tumor (G2–G4) (2); planned elective surgical resection (3); age ≥18 years; and (4) signed informed consent.

The exclusion criteria were (1): presence of other primary or metastatic tumors (2); emergency surgical intervention (3); incomplete neuroimaging; and (4) insufficient tissue quality for molecular analysis.

The preliminary diagnosis of astrocytic tumors was established through contrast-enhanced computed tomography (CT) and further confirmed using magnetic resonance imaging (MRI). The MRI protocol included T1- and T2-weighted sequences, fluid-attenuated inversion recovery (FLAIR) sequences, and, when necessary, diffusion tensor imaging. In cases where the tumor was located near eloquent brain regions, additional imaging techniques such as functional MRI and diffusion MRI tractography were employed to assist with neuronavigation.

Surgical resection aimed for maximal tumor removal while minimizing damage to adjacent healthy tissue. Intraoperative techniques included neuronavigation, fluorescence-guided surgery using 5-aminolevulinic acid (5-ALA) for grade IV tumors, and direct cortical stimulation for lesions in proximity to the sensorimotor cortex. The definitive diagnosis was established through histopathological evaluation, classifying tumor malignancy according to the World Health Organization (WHO) grading system (8–10).

### 2.2 Extraction of total ribonucleic acid

Tissue samples were homogenized using a T18 Digital Ultra-Turrax handheld rotor-stator homogenizer (IKA Poland Ltd., Warsaw, Poland). Total RNA was extracted with TRIzol reagent (Invitrogen Life Technologies, Carlsbad, CA, USA), following the manufacturer’s protocol. To further purify the RNA and remove contaminants, the RNeasy mini kit (QIAGEN, Hilden, Germany) was employed. Additionally, DNase I treatment (Fermentas International Inc., Burlington, ON, Canada) was applied to eliminate any residual genomic DNA.

RNA quality was assessed through 1% agarose gel electrophoresis with ethidium bromide staining (0.5 mg/mL) to verify integrity. RNA concentration was determined by measuring absorbance at 260 nm, ensuring accurate quantification of sample yield and purity.

### 2.3 Microarray analysis of gene expression

A comparative analysis of circadian clock-related gene expression in tumor tissues versus control tissues was conducted using the HG-U 133_A2 microarray platform (Affymetrix, Santa Clara, CA, USA) and the GeneChip™ 3′ IVT PLUS reagent kit (Affymetrix; Catalog Number 902416). The study strictly adhered to the manufacturer’s protocols and methodologies established in previous research (38).

### 2.4 MicroRNA profiling and target prediction

To investigate the role of circadian clock-related microRNAs (miRNAs) and their influence on gene expression, a microarray analysis was performed using the GeneChip miRNA 2.0 Array (Affymetrix). This commercial platform ensures high precision and reliability in detecting differentially expressed miRNAs between tumor and control tissues. The microarray profiling process was conducted in strict accordance with the manufacturer’s instructions to maintain standardization and reproducibility. Identified miRNAs that demonstrated differential expression were further analyzed using two widely recognized databases—TargetScan (http://www.targetscan.org/) (39) and miRanda (http://mirdb.org)— (40) to predict their interactions with messenger RNAs (mRNAs). Predicted targets with a confidence score exceeding 80 were considered highly reliable, indicating strong miRNA–mRNA interactions. Conversely, predictions with scores below 60 required further validation to confirm their authenticity (40, 41).

### 2.5 Validation of gene expression by quantitative reverse-transcription polymerase chain reaction

To validate the microarray data, qRT-PCR was performed on selected genes using the SensiFast SYBR No-ROX One-Step kit (Bioline, London, UK). The thermal cycling protocol included reverse transcription at 45°C, polymerase activation at 95°C for 2 minutes, followed by 40 cycles of denaturation at 95°C for 5 seconds, annealing at 60°C for 10 seconds, and elongation at 72°C for 5 seconds. Gene expression levels were analyzed using the 2^−ΔΔCt^ method, where a fold change of 1 represented the control group, values greater than 1 indicated overexpression, and values below 1 signified gene silencing. To ensure accuracy and consistency, glyceraldehyde 3-phosphate dehydrogenase (GAPDH) was used as an internal control for normalization. Detailed primer sequences are provided in **Table 1** for reference.

**Table 1:** Nucleotide sequence of the primers used in RT-qPCR for TGF-β-1–3 and GAPDH.

mRNA	Oligonucleotide sequence	Tm (°C)
*TGF-β1*	Forward: 5’-GGCCAGATCCTGTCCAAGC-3’ Reverse: 5’-GTGGGTTTCCACCATTAGCAC-3’	85.4
*TGF-β2*	Forward: 5’-CAGCACACTCGATATGGACCA-3’ Reverse: 5’-CCTCGGGCTCAGGATAGTCT-3’	88.7
*TGF-β3*	Forward: 5’-CTGGATTGTGGTTCCATGCA-3’ Reverse: 5’-TCCCCGAATGCCTCACAT-3’	86.6
*GAPDH*	Forward: 5’-GGTGAAGGTCGGAGTCAACGGA-3’ Reverse: 5’-GAGGGATCTCGCTCCTGGAAGA-3’	86.4

Forward, sense primer; reverse, antisense primer; Tm, melting temperature; GAPDH, glyceraldehyde 3-phosphate dehydrogenase; TGF-β-1-3, transforming growth factor beta 1-3. The specificity of RT-qPCR was confirmed by determining the melting temperature for each amplimer.

### 2.6 DNA Methylation analysis by methylation-specific PCR

CpG island locations within the selected gene sequences were identified using the MethPrimer program (http://www.urogene.org/cgi-bin/methprimer/methprimer.cgi; accessed January 19, 2025) (42). Primer design followed strict criteria, including a CpG island length exceeding 100 nucleotides, a GC content above 50%, and an observed-to-expected ratio greater than 0.6 (**Table 2**).

**Table 2:** Characteristics of primers designed for the MSP.

mRNA	M/U	NCBI Reference Sequen	Primers (5′-3′
*TGF-β1*	M	NM_000660.7	Forward: GTAGGATTTGGGGATTTTAGATC Reverse: AAACAAACCGAAAATAAAAACGAC
	U		Forward: GTAGGATTTGGGGATTTTAGATTGT Reverse: AAAAACAAACCAAAAATAAAAACAAC
*TGF-β2*	M	NM_001135599	Forward: GTAGTGGAAGGTAGGATCGAATC Reverse: TATCAATCTCGAATACGAAATAACG
	U		Forward: GTAGTGGAAGGTAGGATTGAATTG Reverse: TCAATCTCAAATACAAAATAACAAA
*TGF-β3*	M	NM_003239.5	Forward: TAATTTATTTCGAGTAGAATTTCGG Reverse: ATAACATCAAAAAACAACCACTCG
	U		Forward: TAATTTATTTTGAGTAGAATTTTGG Reverse: TAACATCAAAAAACAACCACTCAAC

M, primers designed for methylated sequences; U, primers designed for non-methylated sequence; TGF-β-1-3, transforming growth factor beta 1-3.

To assess DNA methylation status, sodium bisulfite conversion was performed according to the manufacturer’s recommendations, followed by sample purification. Methylation-specific PCR (MSP) was carried out using the QuantiTect SYBR Green PCR Kit (Qiagen GmbH, Hilden, Germany) with designed primers. The thermal cycling protocol consisted of an initial denaturation at 95°C for 5 minutes, followed by 40 cycles of 30 seconds each at 94°C (denaturation), 65°C (annealing), and 72°C (elongation).

For further analysis, the methylation status of specific genes, including irisin, ghrelin, and titin, was evaluated by electrophoresis of PCR products on a 1% agarose gel containing ethidium bromide (0.5 µg/mL) in 1x TBE buffer at 120 V. Fragment sizes were assessed using the pBR322/HaeIII size marker. To confirm the specificity of amplification, control samples consisting of methylated and non-methylated DNA were included, utilizing the EpiTect Control DNA set (Qiagen GmbH, Hilden, Germany).

### 2.7 Protein quantification by enzyme-linked immunosorbent assay and western blot

The expression levels of TGF-β-1, TGF-β-2, and TGF-β-3 were quantified using both enzyme-linked immunosorbent assay (ELISA) and Western blot analysis following electrophoretic separation in polyacrylamide gel. Polyclonal antibodies specific to each TGF-β isoform were used according to the manufacturer’s protocols: anti-TGF-β-1 (bs-0086R, STI, Poznań, Poland; dilution 1:1000), anti-TGF-β-2 (bs-20412R, STI, Poznań, Poland; dilution 1:1000), and anti-TGF-β-3 (bs-0099R, STI, Poznań, Poland; dilution 1:1000). Glyceraldehyde-3-phosphate dehydrogenase (GAPDH) (sc-47724, Santa Cruz Biotechnology, Dallas, TX, USA; dilution 1:500) was used as a loading control. For signal detection, a horseradish peroxidase (HRP)-conjugated goat anti-rabbit IgG secondary antibody (Bio-Rad, Milan, Italy; catalog number 1706515; dilution 1:3000) was applied.

Absorbance measurements for ELISA were performed at 540 nm using an M200PRO microplate reader (Tecan, Männedorf, Switzerland). A detailed description of the ELISA and Western blot procedures is available in our previous publications (38).

To validate assay performance, recombinant TGF-β proteins were used as positive controls for each isoform. These included recombinant TGF-β-1 (MBS2122438), TGF-β-2 (MBS2153787), and TGF-β-3 (MBS2086791), all purchased from MyBioSource Inc. (San Diego, CA, USA). A detailed description of the ELISA and Western blot protocols can be found in previous studies (38).

### 2.8 Immunohistochemical detection of TGF-β isoforms

Tissue specimens were sectioned into 8.0 µm-thick slices using a microtome (Leica Microsystems, Germany). The subsequent processing steps, including dehydration, antigen retrieval, antibody incubations, and staining, were performed according to the manufacturer’s instructions provided in the manuals for the DAB Substrate Kit (Peroxidase, HRP; Vector Laboratories, Newark, California, USA) and the IHC-Paraffin Protocol (IHC-P; Abcam plc, Cambridge, UK).

Immunohistochemical reactions were observed and documented using a Nikon Coolpix fluorescent optical system. The cellular localization and quantification of the selected proteins were analyzed through computer-assisted image analysis using ImageJ software. Images were captured from three slides at 200× magnification.

The optical density of DAB reaction products was assessed in the regions where immunohistochemical reactions occurred in response to the selected proteins. This analysis was performed using the IHC-Profiler plug-in within ImageJ software. Additionally, the average percentage of the DAB-stained area was calculated relative to the background values in each field.

### 2.9 Statistical analysis

Statistical analyses were conducted using StatPlus and the Transcriptome Analysis Console (Affymetrix). The Shapiro–Wilk test assessed data normality (*p* < 0.05). For group comparisons, one-way ANOVA with Benjamini–Hochberg correction and Scheffe’s *post hoc* test (*p* < 0.05) were applied. Pairwise comparisons were performed using Student’s t-test. Survival analysis was conducted using the Kaplan-Meier method, with differences assessed by the log-rank test. Cox proportional hazards regression evaluated the impact of TGF-β-1 and TGF-β-3, and selected miRNAs expression on survival. All tests were two-tailed, with *p* < 0.05 considered statistically significant.

### 2.10 Sample size analysis

According to national statistics, approximately 3,270 primary brain tumors were diagnosed in Poland between 2020 and 2021 (43). Given our study duration and access to clinical material, we enrolled 65 patients. Assuming a maximum variance (fraction size = 0.5) and a 95% confidence level, this sample yields a maximum margin of error of approximately 12%.

## 3 Results

### 3.1 Microarray and RTqPCR expression analysis of TGF-β-1–3 in G3/G4 astrocytic tumor samples compared to G2 samples

Microarray and RT-qPCR analyses revealed distinct expression patterns of TGF-β isoforms across tumor grades. Microarray data (**Table 3**) showed that TGF-β-1 expression was elevated 1.43-fold in G3 tumors compared to G2 (p < 0.05) and 1.54-fold in G4 tumors compared to G2 (p < 0.05). TGF-β-3 expression showed even greater upregulation, with a 2.76-fold increase in G3 (p < 0.05) and a 3.45-fold increase in G4 samples relative to G2 (p < 0.05). In contrast, TGF-β-2 expression remained relatively stable, with fold changes of 1.10 in G3 and 1.17 in G4, showing no statistically significant differences (p>0.05).

**Table 3:** Microarray profile of *TGF-β-1–3* expression in G3 and G4 astrocytic tumor samples compared to G2 samples.

mRNA	ID	G3 vs. G2	G4 vs. G2
***TGF-β-1***	203085_s_at	1.43 ± 0.21*	1.54 ± 0.54*
***TGF-β-2***	220407_s_at	1.10 ± 0.19	1.17 ± 0.37
***TGF-β-3***	209747_at	2.76 ± 0.53*	3.45 ± 0.46*

Data are presented as mean ± standard deviations; *TGF-β-1-3*, transforming growth factor 1-3, *statistically significance differences (p < 0.05).

**Figure 1:**
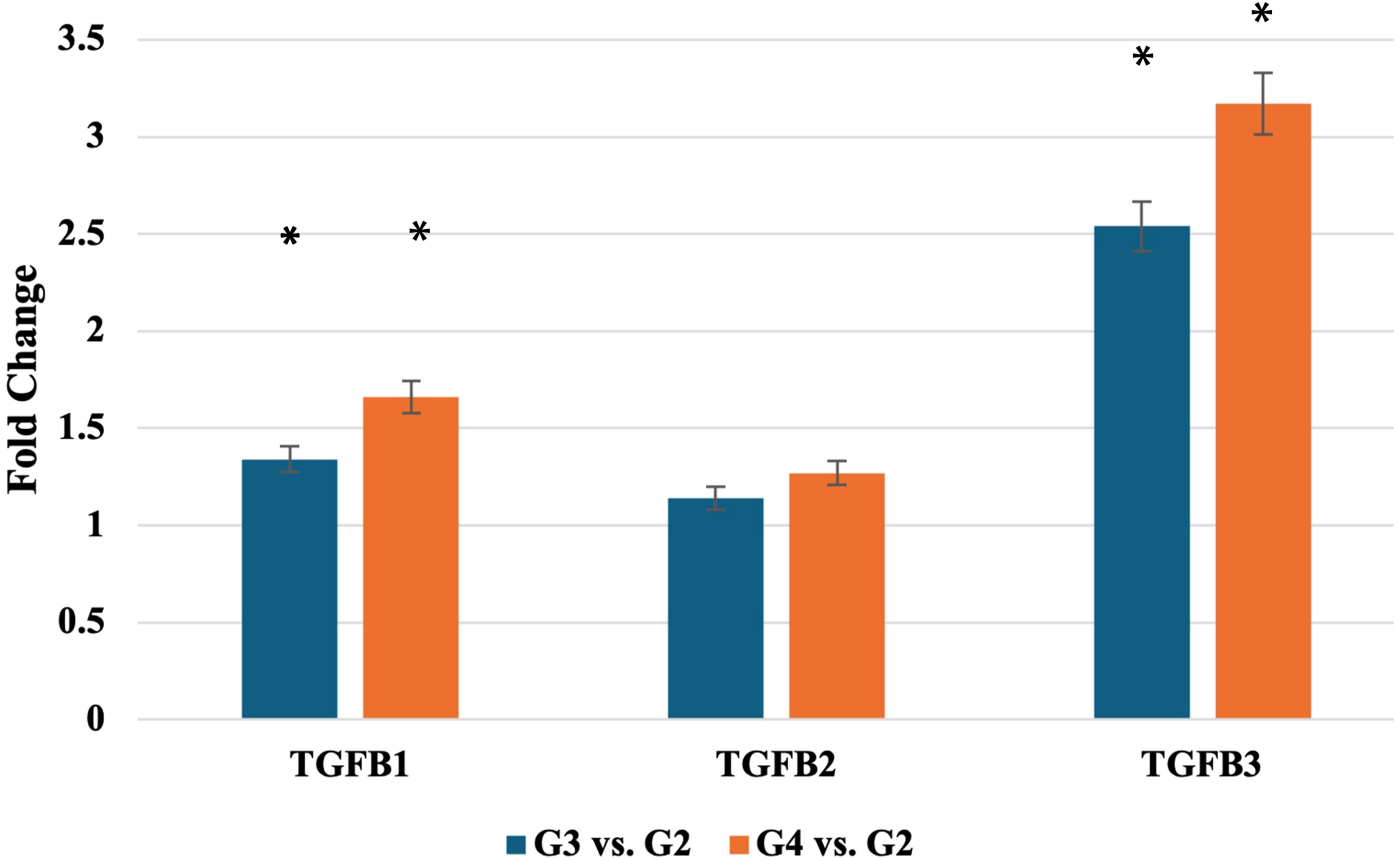
Expression profile of *TGF-β-1–3* mRNA obtained via RTqPCR. TGF-β-1-3, transforming growth factor 1-3; *statistically significance differences (p < 0.05).

RT-qPCR validation (**Figure 1**) confirmed these findings, with TGF-β-1 mRNA levels significantly elevated in G3 and G4 tumors (p < 0.05). Similarly, TGF-β-3 mRNA levels increased significantly in higher grades, while TGF-β-2 mRNA expression did not differ significantly across grades.

These grade-associated increases in *TGF-β-1* and *TGF-β-3* mRNA expression suggest a role in tumor progression. To identify upstream regulators potentially driving these changes, we next analyzed candidate microRNAs with predicted binding to the TGF-β isoforms.

### 3.2 Quantification of TGF-β-1–3 protein concentration in G2, G3, and G4 astrocytic tumors by ELISA and western blot analysis

The ELISA-based quantification of TGF-β-1–3 protein levels in G2, G3, and G4 astrocytic tumors revealed significant differences between tumor grades (**Table 4**). TGF-β-1 concentration increased from 723.87 ± 123.13 pg/ml in G2 to 956.13 ± 87.65 pg/ml in G4, while TGF-β-3 levels rose from 876.23 ± 76.87 pg/ml in G2 to 1108.81 ± 156.73 pg/ml in G4. In contrast, TGF-β-2 remained below the detection limit in all samples.

**Table 4:** Quantification of TGF-β-1–3 Protein Concentration in G2, G3, and G4 Astrocytic Tumors by ELISA.

Isoform of TGF-β	Grading	Concentration [pg/ml]	95%Cl
TGF- β1	2	723.87 ± 123.13	647.55-800.19
	3	876.81 ± 108.19	809.75-943.87
	4	956.13 ± 87.65	901.80-1010.46
TGF- β2	2	< below detection	–
	3	< below detection	–
	4	< below detection	–
TGF- β3	2	876.23 ± 76.87	828.17-923.67
	3	1098.23 ± 219.87	961.12-1234.51
	4	1108.81 ± 156.73	1011.67-1205.95

TGF- β1-3, transforming growth factor beta 1-3; p, value of statistical significance; Data were presents as mean ± standard deviation; 95%Cl, 95% confidence interval; 95% confidence interval); VAS, visual analogue scale.

Statistical analysis revealed a significant main effect (p < 0.05), with *post hoc* testing confirming significant differences between G2 and G4 for both TGF-β-1 and TGF-β-3.

**Figure 2:**
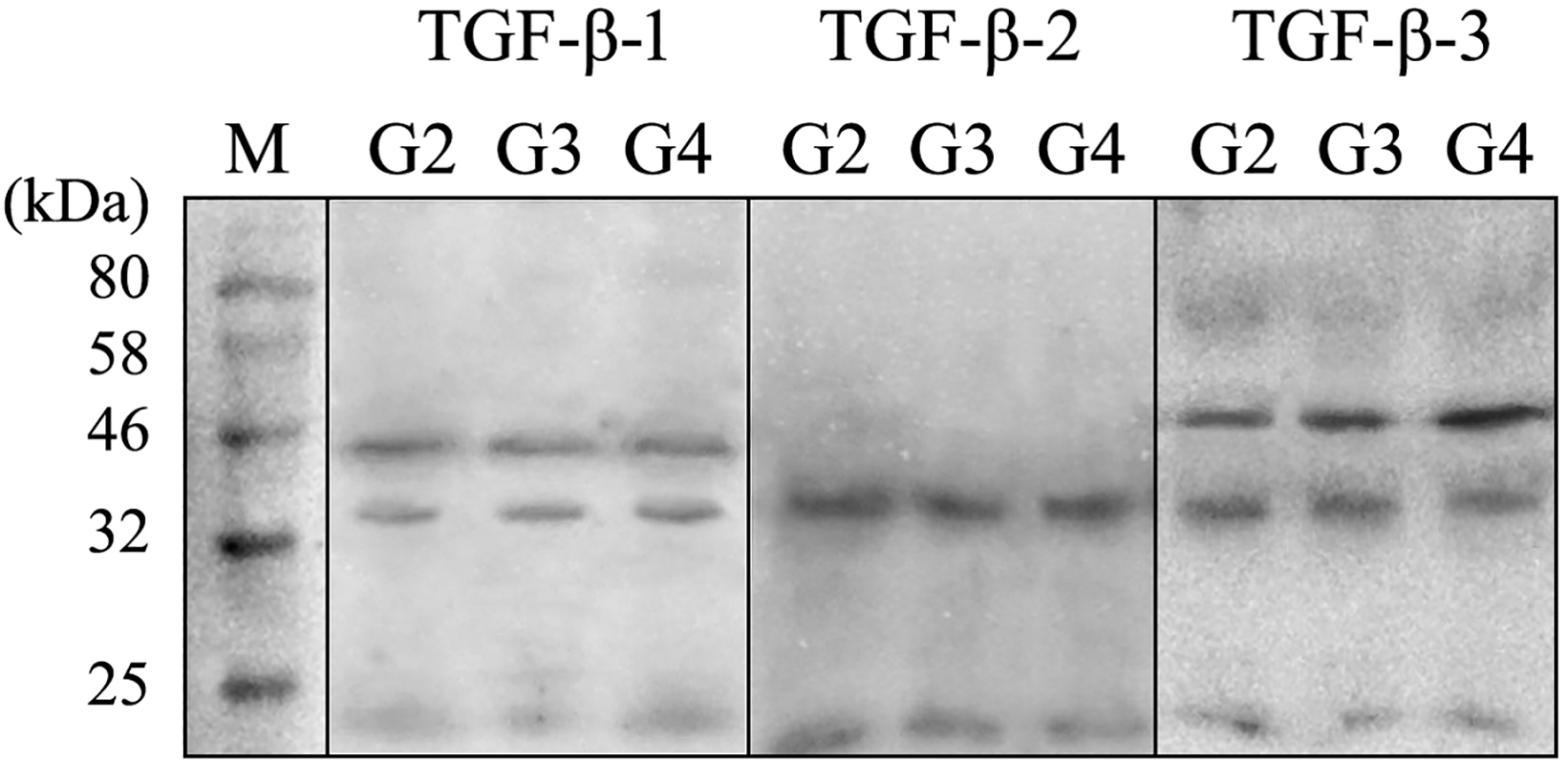
Example of electrophoretic separation of TGF- β1–3 in the G2, G3, and G4 astrocytic tumor samples. TGF β-1 and - 3, transforming growth factor beta 1 and 3; kDa, kilo Daltons; M, size marker (New England Biolabs Marker).

**Figure 2** presents a representative electropherogram, confirming the specificity of the reaction and the integrity of the samples. The presence of TGF-β-1 was validated by detecting a 44 kDa band, while TGF-β-3 was confirmed by a 47 kDa band. The sample quality was further verified by the presence of GAPDH at 37 kDa, serving as a loading control. Western blot quantification showed that TGF-β-1 levels, normalized to GAPD, were 0.34 ± 0.11 in G2 samples, 0.45 ± 0.18 in G3, and 0.47 ± 0.12 in G4, with no significant differences observed (p > 0.05). Notably, TGF-β-2 was not detected in any of the analyzed samples. In contrast, TGF-β-3 expression, also normalized to GAPDH, was significantly elevated across tumor grades, increasing from 2.17 ± 0.42 in G2 to 4.54 ± 0.65 in G3 and 5.15 ± 1.09 in G4 (p < 0.05).

### 3.3 TGF-β-1 and TGF-β-3 expression profile determined by IHC

**Figure 3:**
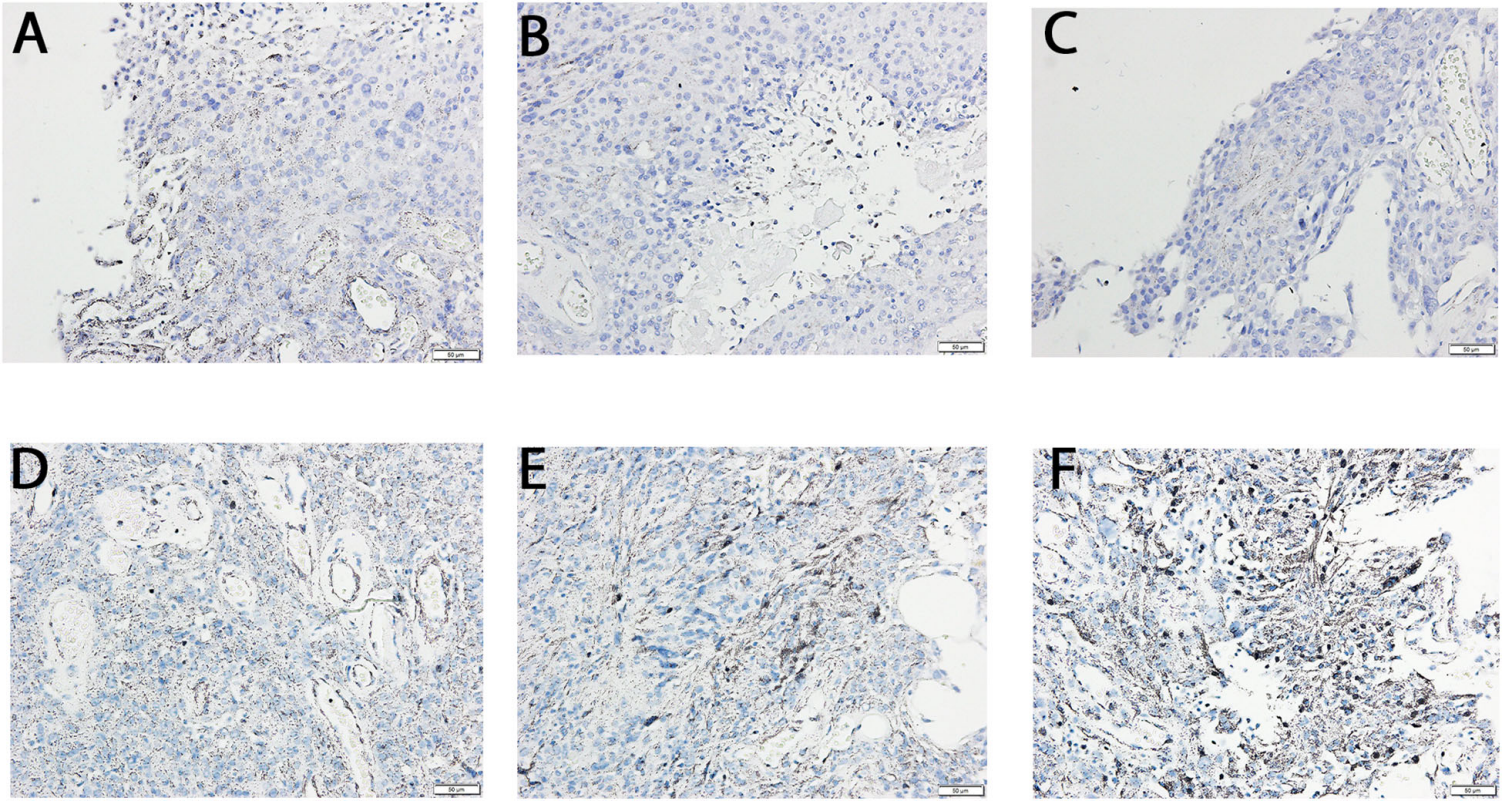
Immunochemical expression of TGF-β -1and -3 in the G2, G3, and G4 astrocytic tumor samples. **(A)** TGF β -1 in G2 samples; **(B)** TGF β -1 in G3 samples; **(C)** TGF β -1 in G4 samples; **(D)** TGF β -3 in G2 samples; E, TGF β -3 in G3 samples; F, TGF β -3 in G4 samples.

IHC staining showed elevated optical density for TGF-β-1 in G3 (137.81 ± 8.76% vs. G2) and G4 (178.01 ± 12.19% vs. G2) samples (p < 0.05). TGF-β-3 optical density increased even more markedly: 195.03 ± 14.56% in G3 and 276.19 ± 19.76% in G4 relative to G2 (p < 0.05). No TGF-β-2 signal was detected in any group. The results were presented in the **Figure 3**.

### 3.4 Predicted miRNA-mediated regulation of TGF-β-1–3 expression

To explore post-transcriptional regulatory mechanisms, we analyzed differentially expressed miRNAs across tumor grades and predicted their targets (**Table 5**). miR-3196 was markedly downregulated in both G3 and G4 astrocytomas and was predicted to target TGF-β-1 (target score: 80). Conversely, miR-466, miR-141-3p, and miR-200a-3p were upregulated and predicted to regulate TGF-β-2 (target scores: 99, 95, and 95, respectively). miR-2278, moderately upregulated in higher-grade tumors, showed a strong predictive association with TGF-β-3 regulation (target score: 92).

**Table 5:** Differential expression profile of miRNAs predicted to regulate TGF-β isoforms in G3 and G4 astrocytic tumor samples compared to G2.

mRNA	miRNA	Target score	log_2_ Fold change (G3 vs. G2)	log_2_ Fold change (G4 vs. G2)
*TGF-β-1*	hsa-miR-3196	80	-3.47 ± 0.34*	-4.56 ± 0.32*
*TGF-β-2*	hsa-miR-141-3p	99	2.89 ± 0.45*	3.12 ± 0.19*
	hsa-miR-200a-3p	95	2.71 ± 0.81*	3.45 ± 0.54*
	hsa-miR-466	95	2.19 ± 0.23	2.76 ± 0.71
*TGF-β-3*	hsa-miR-2278	92	2.54 ± 0.45	2.96 ± 0.23

Data are presented as mean ± standard deviations; *TGF-β-1-3*, transforming growth factor 1-3, *statistically significance differences (p < 0.05).

The predicted interactions between these miRNAs and TGF-β isoforms highlight a potential post-transcriptional mechanism of regulation. To further investigate additional layers of control, we examined the methylation status of these genes across tumor grades.

### 3.5 Epigenetic studies revealed differential DNA methylation patterns of TGF-β isoforms in G2, G3 and G4 tumours

**Figure 4:**
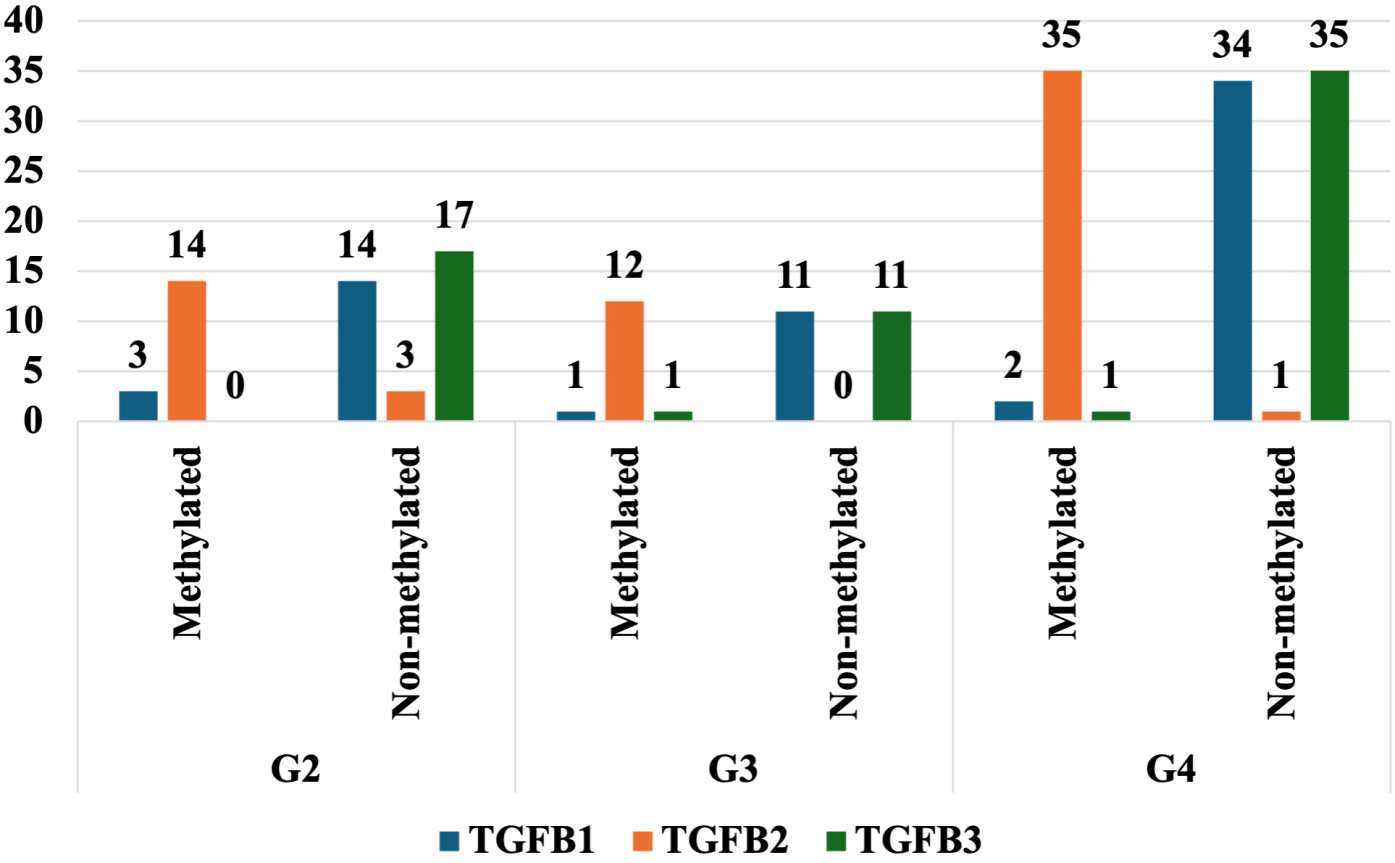
The degree of methylation of selected genes in the G2, G3, and G4 astrocytic tumor samples.

To explore the epigenetic regulation of TGF-β- isoforms in glioma, the DNA methylation patterns of TGF-β–1–3 were analyzed by MSPCR. MSPCR revealed variable epigenetic regulation of TGF-β isoforms by tumor grade. In G2 tumors, partial methylation was observed for TGF-β-1 and TGF-β-3, while TGF-β-2 was unmethylated. In G3 samples, most TGF-β-1 and TGF-β-2 genes were methylated, whereas TGF-β-3 exhibited low methylation. In G4 tumors, TGF-β-2 was highly methylated in nearly all cases, while TGF-β-1 and TGF-β-3 showed mixed methylation statuses (**Figure 4**).

### 3.6 Kaplan-Meier survival analysis and cox proportional hazards model for TGF-β-1 and TGF-β-3 in astrocytic tumors

**Figure 5:**
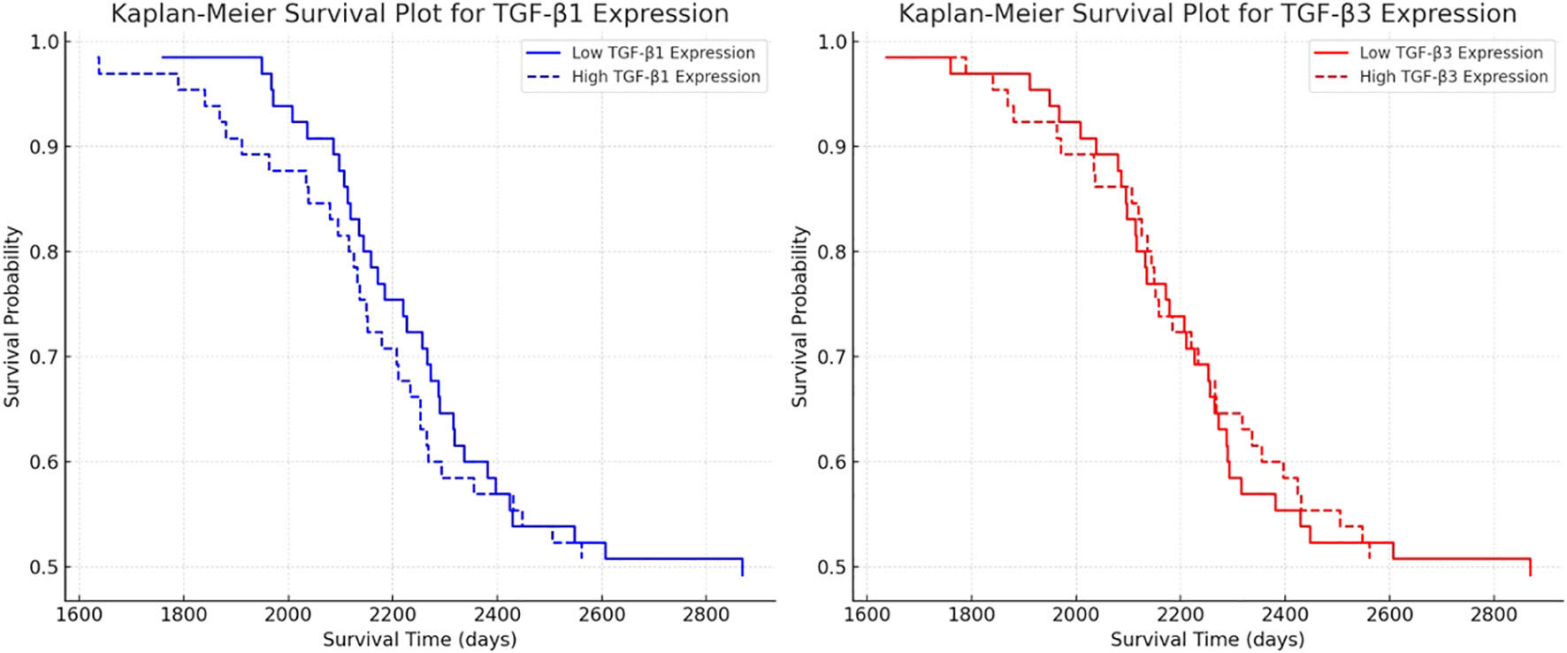
Kaplan-Meier survival analysis for TGF-β-1 and TGF-β-3 expression in astrocytic tumors.

Kaplan-Meier survival analysis was conducted to evaluate the prognostic impact of TGF-β-1 and TGF-β-3 expression on patient survival, revealing that higher expression levels of both isoforms were associated with poorer survival outcomes (**Figure 5**). Cox regression analysis for TGF-β-1 (*Exp(B) = 1.00225, p = 0.08054*) indicated that patients with higher TGF-β-1 expression exhibited reduced survival, but the effect was not statistically significant (*p* > 0.05), suggesting that while a trend was observed, additional validation may be required to confirm its prognostic relevance. In contrast, TGF-β-3 expression (*Exp(B) = 1.02644, p < 0.0001*) was strongly associated with decreased survival, with each unit increase in expression raising the risk of death by 2.6%. This effect was highly statistically significant (*p* < 0.0001), indicating that TGF-β-3 could serve as a valuable prognostic biomarker in astrocytic tumors. Overall, these findings suggest that TGF-β-3 expression plays a crucial role in tumor progression and patient survival, whereas TGF-β-1 exhibits a weaker, albeit negative, influence on survival outcomes.

**Figure 6:**
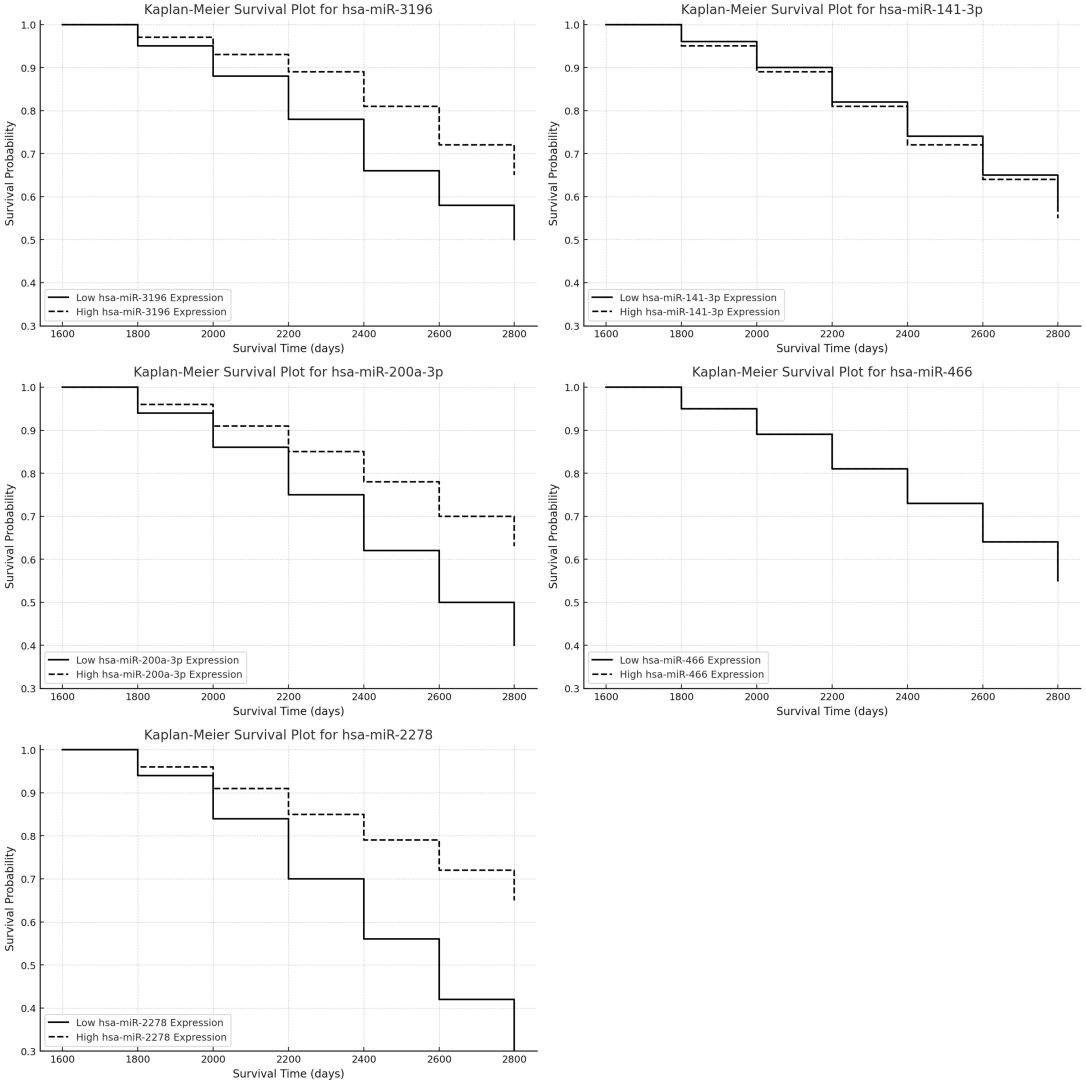
Kaplan-Meier survival analysis for selected miRNAs expression in astrocytic tumors.

In turn, Kaplan-Meier survival analysis was conducted to evaluate the prognostic impact of hsa-miR-3196, hsa-miR-141-3p, hsa-miR-200a-3p, hsa-miR-466, and hsa-miR-2278 expression on patient survival, revealing that higher expression of hsa-miR-2278 and lower expression of hsa-miR-3196 were associated with poorer survival outcomes (**Figure 6**). Cox regression analysis for hsa-miR-3196 (Exp(B) = 0.8897, *p* = 0.076) indicated that patients with reduced expression exhibited decreased survival, though the effect did not reach statistical significance (*p* > 0.05), suggesting a negative survival trend that warrants further investigation. In contrast, hsa-miR-2278 (Exp(B) = 1.437, *p* = 0.008) was significantly associated with increased mortality risk, with each unit increase in expression elevating the risk of death by approximately 43.7%. This effect was statistically significant (*p* < 0.01), supporting the potential of hsa-miR-2278 as a prognostic biomarker in astrocytic tumors. The remaining miRNAs—hsa-miR-141-3p, hsa-miR-200a-3p, and hsa-miR-466—showed no significant impact on survival, indicating limited prognostic value in this cohort.

## 4 Discussion

Despite increasing recognition of TGF-β’s role in glioma biology, the distinct contributions of its isoforms (TGF-β-1, -β2, and -β3) across tumor grades remain unclear. Our study addresses this critical gap by integrating expression profiling, DNA methylation, and miRNA regulation to elucidate the isoform-specific dynamics and prognostic significance of TGF-β in astrocytic tumors. Our results demonstrate significantly increased expression of TGF-β-1 and TGF-β-3 in G3 and G4 tumors compared to grade II (G2), while TGF-β-2 levels remained statistically unchanged. These findings align with prior studies reporting upregulation of TGF-β isoforms in high-grade gliomas. According to other researchers, including Frei et al., there is a strong correlation between TGF-β isoform expression and glioma progression [51]. Their study analyzed 64 newly diagnosed and 16 recurrent astrocytic tumors, assessing TGF-β-1 to TGF-β-3 expression. Among the isoforms, *TGF-β-1* mRNA was the most abundant, while *TGF-β-3* mRNA was the least. Survival analysis indicated that patients with high mRNA expression of *TGF-β-2* or pSmad1/5/8 protein had worse outcomes compared to other study participants (44).

Kurowska et al. investigated 43 astrocytic tumor tissue sections across various disease stages, quantitatively assessing TGF-β isoform mRNA levels and the expression of genes associated with TGF-β signaling (22). Their microarray analysis showed a statistically significant increase in *TGF-β-1* and *TGF-β-2* expression in G3/G4 tumors compared to G2, whereas RT-qPCR validation confirmed this increase only for *TGF-β-2*. The authors suggested that quantifying *TGF-β-2* mRNA could be a valuable diagnostic tool in the future (22). This finding differs from our study results, which indicate that *TGF-β-3* is a strong prognostic biomarker in astrocytic tumors, significantly correlating with poor survival outcomes. *TGF-β-1* showed an increasing trend in high-grade tumors but lacked statistical significance in survival analysis. These results highlight *TGF-β-3* as a potential therapeutic target in astrocytic tumors. Differences in study outcomes may arise from methodological variations, patient cohort composition and size, or tumor microenvironment-specific factors. The roles of individual TGF-β isoforms in gliomas may be more complex than previously thought, warranting further investigation into the regulatory mechanisms of these cytokines in tumor progression.

A study by Battle et al. demonstrated that genetic and epigenetic changes can alter transcriptional circuits in cancer cells, leading to a shift in TGF-β signaling from protective to tumor-promoting effects (45). This study highlights that TGF-β-mediated immune suppression is context-dependent. Battle et al. state that in tissues exposed to continuous antigenic stimulation, such as the gastrointestinal tract, TGF-β suppresses inflammation and adaptive immune responses, thereby reducing tumor development. However, in advanced cancer stages, TGF-β signaling enables immune evasion in various tumor types and contributes to disease progression (45). This effect allows transformed tumor cells to evade immune system-mediated destruction, including by T cells and natural killer (NK) cells. Understanding TGF-β’s role in cancer development provides hope for identifying specific TGF-β pathway inhibitors to improve cancer prognosis.

Our study demonstrates the significant impact of epigenetic factors on the regulation of TGF-β-1–3 expression in astrocytic tumors. We observed different methylation patterns across various tumor grades. In G2 tumors, methylation was detected for TGF-β-1 and TGF-β-3 transcripts, whereas TGF-β-2 remained unmethylated. In G3 astrocytic tumors, most samples showed methylation for TGF-β-1 and TGF-β-2, whereas TGF-β-3 exhibited minimal methylation. In G4 tumors, nearly all samples were methylated for TGF-β-2, whereas TGF-β-1 and TGF-β-3 displayed a mixture of methylated and unmethylated cases. These observed methylation trends suggest potential epigenetic regulation of TGF-β expression in astrocytic tumor progression.

In our study, we identified several miRNAs that may influence the regulation of TGF-β expression. MiR-3196 was found to be significantly downregulated in G3 and G4 tumors, with predictive analysis indicating its potential role in regulating TGF-β-1 expression. Notably, hsa-miR-466, hsa-miR-141-3p, and hsa-miR-200a-3p were upregulated in both tumor grades, with high predictive scores for regulating TGF-β-2. Similarly, hsa-miR-2278, which showed moderate upregulation, demonstrated a strong predictive association with TGF-β-3 regulation.

Qi-Qi et al. highlight the role of miR-141, which functions as either a tumor suppressor in different cancers, regulating tumor cell proliferation, apoptosis, invasion, and metastasis through various signaling pathways, including the phosphatidylinositol-3-kinase (PI3K)/protein kinase B (AKT) pathway and the constitutive activation of nuclear factor-κB (NF-κB). Their validation of the target gene and pathway analysis provided insights into the role of this miRNA in different tissues (46). Their review also presents new findings suggesting that miR-141 could serve as a non-invasive biomarker and therapeutic target for several types of cancer.

A meta-analysis by Peng et al. on the miR-200 family demonstrated that these regulatory epigenetic molecules modulate physiological and pathological processes by targeting multiple genes, ultimately influencing glioma cell proliferation and invasion, as well as therapeutic response and prognosis (47). In turn, the study by Chen et al. reported that grade IV glioma tissues exhibited significantly lower levels of miR-200a compared to grade II–III gliomas. Glioma patients were categorized into high- and low-miR-200a expression groups based on the median expression value. Their analysis showed that low miR-200a expression was significantly associated with an advanced clinical stage of glioma. Additionally, miR-200a levels were markedly reduced in several common glioma cell lines compared to healthy astrocytic cells (48). The research by Bian et al. demonstrated that miR-141 was significantly downregulated in glioma tissues and cell lines compared to normal brain tissues, with its expression correlating with pathological tumor grade. Forced expression of miR-141 in glioma cells significantly inhibited cell proliferation, migration, and invasion, whereas miR-141 silencing had the opposite effect (49).

In summary, our study revealed a complex and isoform-specific relationship between promoter methylation, microRNA expression, and the regulation of TGF-β isoforms in astrocytic tumors. Notably, TGF-β-2 exhibited substantial promoter methylation in glioblastoma (G4) samples but did not show consistent downregulation at the mRNA or protein level, suggesting that promoter methylation alone is insufficient to repress its expression in this context. This finding implies the involvement of compensatory or overriding regulatory mechanisms such as miRNA-mediated translational control. In contrast, TGF-β-3 expression was significantly upregulated in high-grade tumors despite exhibiting relatively low promoter methylation, indicating methylation-independent transcriptional activation. Our integrated analysis of differentially expressed miRNAs further supports this regulatory complexity. Specifically, the downregulation of miR-3196, predicted to target TGF-β-1, and upregulation of miR-2278, predicted to regulate TGF-β-3, were both associated with increased expression of their respective protein targets. Meanwhile, miRNAs predicted to target TGF-β-2 (e.g., hsa-miR-141-3p and hsa-miR-200a-3p) were found to be upregulated in higher-grade tumors, potentially contributing to post-transcriptional repression of TGF-β-2, thereby explaining the discrepancy between methylation and expression profiles. These findings are consistent with the concept of mutual regulation between miRNAs and DNA methylation in cancer epigenetics, as described by Wang et al. (50). In this model, miRNAs can influence the activity of DNA methyltransferases (DNMTs), while their own expression is in turn modulated by promoter methylation. Thus, in gliomas, the interplay between miRNA expression and DNA methylation may create a dynamic regulatory network governing the expression of TGF-β isoforms. This dual-layered control appears to be grade-dependent, emphasizing the importance of considering both epigenetic mechanisms in understanding glioma progression and in identifying therapeutic targets (31, 50–54).

Furthermore, in our study, we assessed the correlation between TGF-β-1 and TGF-β-3 expression and five-year survival. Our survival analysis indicated that high TGF-β-3 expression was significantly associated with poor prognosis, whereas the effect of TGF-β-1 on survival was weaker and did not reach statistical significance. This finding aligns with previous reports suggesting that TGF-β plays a complex role in astrocytic tumor progression, acting as a tumor suppressor in the early stages and a promoter of aggressiveness in advanced cases (55, 56).

Elevated TGF-β-3 expression in high-grade tumors may contribute to a more invasive tumor phenotype through several mechanisms. Firstly, TGF-β is a key regulator of epithelial-to-mesenchymal transition (EMT), which enhances the migratory and invasive capabilities of cancer cells. In gliomas, this process facilitates tumor spread within the brain, leading to poorer prognosis (57). secondly, TGF-β exerts immunosuppressive effects by inhibiting T and NK cell activity and promoting the M2 phenotype of suppressor cells in the tumor microenvironment, thereby reducing the efficacy of the body’s immune response (24, 26, 27).

Moreover, our Cox regression analysis revealed that each unit increase in TGF-β-3 expression elevated the risk of patient mortality by 2.6% (Exp(B) = 1.02644, p < 0.0001), highlighting its strong prognostic significance. While high TGF-β-1 expression also trended toward worse survival outcomes, this association did not reach statistical significance (Exp(B) = 1.00225, p = 0.08054). It is possible that TGF-β-1 plays a more complex role, with its net effect on glioma progression depending on molecular context and interactions with other signaling pathways.

The strong correlation between elevated TGF-β-3 expression in high-grade tumors and poor patient survival suggests its potential use as a biomarker for risk stratification and therapeutic decision-making. Current treatment strategies for high-grade gliomas primarily involve surgical resection, radiotherapy, and temozolomide-based chemotherapy. Unfortunately, these approaches have limited efficacy, and gliomas often develop resistance to treatment (58). Increased TGF-β-3 expression may contribute to this resistance by promoting immunosuppression and angiogenesis in the tumor microenvironment, suggesting that targeted inhibition of TGF-β-3 could enhance the effectiveness of existing therapies.

Although pharmacologic inhibition of the TGF-β pathway has long been explored in glioma, clinical applications have been hampered by systemic toxicity and the pleiotropic, context-dependent nature of TGF-β signaling (59, 60). Direct inhibition of TGF-β can disrupt its tumor-suppressive functions in early-stage disease while only partially suppressing its pro-oncogenic effects in advanced stages. In this context, the microRNAs identified in our study—particularly miR-2278 and miR-3196—may provide a more selective and biologically nuanced means of modulating the TGF-β pathway. Because miRNAs act post-transcriptionally and, in a tissue,-specific manner, they may allow for isoform- and context-selective regulation with fewer off-target effects compared to systemic inhibitors (61–63). These miRNAs not only reflect key aspects of tumor biology but also represent promising therapeutic targets for overcoming TGF-β–driven progression and resistance in astrocytic tumors.

In addition to gene-level analysis, we evaluated the prognostic impact of selected regulatory microRNAs predicted to target TGF-β isoforms. Kaplan-Meier survival analysis revealed that high expression of hsa-miR-2278 was significantly associated with worse patient outcomes, with a 43.7% increase in mortality risk per unit expression (Exp(B) = 1.437, p = 0.008). This finding is particularly relevant given that miR-2278 is predicted to regulate TGF-β-3, suggesting a mechanistic link between its upregulation in high-grade gliomas and the enhanced expression of this profibrotic cytokine. Conversely, hsa-miR-3196 exhibited a trend toward better survival in the high-expression group (Exp(B) = 0.8897, p = 0.076), consistent with its downregulation in high-grade tumors and putative targeting of TGF-β-1. Other analyzed miRNAs (hsa-miR-141-3p, hsa-miR-200a-3p, and hsa-miR-466) did not show statistically significant associations with survival. These results suggest that miR-2278 and miR-3196 may influence glioma progression via post-transcriptional regulation of TGF-β isoforms and may serve as potential biomarkers for patient stratification.

The upregulation of TGF-β-3 in high-grade gliomas may drive a more aggressive phenotype via several mechanisms. Current treatment strategies for high-grade gliomas primarily involve surgical resection, radiotherapy, and temozolomide-based chemotherapy. Unfortunately, these approaches have limited efficacy, and gliomas often develop resistance to treatment (58). Increased TGF-β-3 expression may contribute to this resistance by promoting immunosuppression and angiogenesis in the tumor microenvironment, suggesting that targeted inhibition of TGF-β-3 could enhance the effectiveness of existing therapies. Similarly, modulation of TGF-β-regulating miRNAs, such as miR-2278 and miR-3196, may offer novel therapeutic avenues for influencing tumor behavior and overcoming resistance mechanisms.

This study highlights the differential expression and epigenetic regulation of TGF-β isoforms in astrocytic tumors, demonstrating that TGF-β-1 and TGF-β-3 exhibit increased expression in high-grade tumors, whereas TGF-β-2 remains largely unchanged. Notably, TGF-β-3 emerged as a strong prognostic biomarker, significantly correlating with poorer survival, whereas TGF-β-1 showed an increasing trend in high-grade tumors but lacked statistical significance in survival analysis. The observed methylation patterns and miRNA interactions suggest a complex epigenetic regulation of TGF-β signaling in tumor progression, further emphasizing its role in glioma pathophysiology. These findings underscore the potential of TGF-β-3 as a therapeutic target, highlighting the need for further studies to explore targeted interventions aimed at modulating its expression and activity. While this study provides important molecular insights, future research integrating functional assays and larger patient cohorts is necessary to validate these results and advance precision medicine approaches in astrocytic tumor management.

### 4.1 Limitations of the study

While this study offers meaningful insights into the differential expression and epigenetic regulation of TGF-β isoforms and their associated microRNAs across astrocytic tumor grades, several limitations should be considered. First, the modest sample size (n = 65) from a limited geographical region may restrict the generalizability of the findings and introduces the potential for selection bias. Although TGF-β-3 emerged as a strong prognostic marker, the novelty of the study is tempered by existing literature extensively documenting the role of TGF-β signaling in glioma (22, 23, 64–70). Moreover, although predictive bioinformatics analyses identified candidate miRNAs and methylation patterns potentially regulating TGF-β isoforms, the lack of functional validation through *in vitro* assays (e.g., miRNA modulation, dual-luciferase reporter assays, or SMAD phosphorylation studies) limits the mechanistic interpretation. Similarly, interactions between treatment history, genetic markers such as IDH mutation, and the expression of TGF-β isoforms were not explored. Despite these limitations, the study provides a comprehensive, multi-level molecular profile of TGF-β isoforms in glioma and identifies TGF-β-3 and miR-2278 as promising prognostic indicators. Future studies should incorporate functional experiments, broader patient cohorts, and multivariate analyses to confirm these findings and support their clinical relevance.

## Data Availability

The original contributions presented in the study are publicly available. This data can be found here: https://doi.org/10.6084/m9.figshare.29304938.v1.
